# Annotations

**Published:** 1904-05-07

**Authors:** 


### Annotations.

### Optical Organisation.

There are increasing evidences that certain persons engaged in the trade of selling spectacles are determined to attain a style and dignity which will be interpreted by the public as an official guarantee of their ability to treat defects of vision. We have long been familiar with modest protests of this order set forth in conspicuous letters in individual shop windows and sustained by the claims of peculiar skill and special material. This genial custom has recently been dignified, in some instances, by the assumption of the term "optologist" and by the display of a "diploma." Even this, it appears, will not satisfy the ambition of certain members of the craft. These now desire to restrict the practice of supplying spectacles to the public to their noble selves. Their souls are grieved within them to find that opticians, jewellers, chemists and others, who have not received the peculiar virtue that attaches to an "optologist," are as free as themselves to sell spectacles to all who wish to buy them. Hence a proposal of restrictive legislation and of the institution of compulsory registration. The Worshipful Company of Spectacle-makers it seems already has a register to which goldsmiths dealing in spectacles are admitted after examination, certain parts of the examination being excused to those who have been in business for seven years. This apparently has excited certain chemists who sell spectacles, and consequently a chemist-opticians' association is to come into existence, and a claim is advanced that the members of this must be treated in regard to examinations not less generously than the jewellers. The *Pharmaceutical Journal* goes still further and demands partial exemption for all chemists "simply because they are chemists," and have already "gone over some of the ground." The policy of the chemist, it seems, is to obtain "the diploma of whichever of the recognised examining bodies is prepared to give concessions to the craft as a whole." It is in this way that business instincts arrange to combine the love of science and the sale of spectacles with a minimum of examination.

### Clinical Picture Galleries.

The developments during recent years of the art of illustration have exercised on medical, as on other forms of literature, a very decided influence. Not only do we now get well-illustrated text-books and manuals, but numerous atlases portraying in a highly successful fashion the manifestations and results of disease appear at not infrequent intervals. The interest attaching to many of these is considerable, but it may be questioned whether their utility for purposes of clinical instruction is fully appreciated. It is comparatively rare in our medical schools to see anything like a well-framed attempt to use existing illustrations for the purpose of aiding ordinary ward teaching. This is the more strange seeing that the opportunities for such an attempt are so numerous, and seeing, also, that the student's experience must in the nature of things present many and considerable gaps. The truth seems to be that the value of illustrations as a means of supplementing clinical teaching is but rarely recognised. In addition, it must be allowed that the search for suitable pictures through a number of clumsily-constructed atlases is often an exhausting process, and the risk of damage or destruction which is entailed by their exhibition is a considerable one. Now all these inconveniences can be avoided by the organisation of a clinical picture gallery. For this purpose there is already abundant material in existence, but so long as this remains stowed away in libraries it does but little good, for it is practically inaccessible. If, on the other hand, a carefully selected series of pictures were glazed and mounted and arranged on the walls of a suitable room a most valuable means of clinical instruction would be secured. Much space at our medical schools is occupied by pathological museums, and no one would propose to curtail this. But it is not less important that the clinical training of the student should be made as extensive as possible, and as a complete range of observations is not practicable during the comparatively brief period of the medical curriculum, every method by means of which these can be supplemented should be brought into operation. One such method is the organisation of a clinical picture gallery, and it would be a distinct gain were the need for this recognised in every medical school.

### The Pathology of Heart Disease.

Although a due recognition of the interdependence of the organs of the body has been one of the leading features of medical progress in the course of the last century, much enlightenment is still required in this direction for a proper and full discrimination of cause and effect, for a clear distinction between the primary seat of disease and its secondary manifestations. The problem, moreover, presents itself in considering not only the system as a whole but also the component parts of each individual organ. In the case of the heart, for example, it is doubtful if sufficient attention is devoted even at the present day to diseased conditions of the myocardium. Lesions of the valves or pericardium are so much more easily appreciated at post-mortem examinations than inflammatory or degenerative changes in the heart muscle that the latter are apt to receive too little attention from the medical practitioner. But the fault lies with the clinician and not with the pathologist, for much good work has been done by the latter in elucidating the disease processes to which the muscular apparatus of the heart is liable. It is curious to note, therefore, that the reverse is true with respect to disturbances of the cardiac nervous system. "Neurotic heart" is not at all an infrequent bedside diagnosis, but a minute study of the cardiac nerves in a large number of cases dying of heart disease, whether "organic" or otherwise, is yet required towards completing our knowledge of the pathology of the circulatory system and suggesting lines for diagnosis and treatment. Now that the degenerative changes in nerve cells and their processes can be so well observed by special microscopic methods such an inquiry may be expected to yield most useful information, whether it tends to prove that nerve derangements are more frequent or less frequent causes or concomitants of cardiac disturbance than we at present suppose. At any rate, knowledge is what we require, and here are facts waiting to be collected and presented for our future guidance.

